# PI3K/Akt inhibitor partly decreases TNF-α-induced activation of fibroblast-like synoviocytes in osteoarthritis

**DOI:** 10.1186/s13018-019-1394-4

**Published:** 2019-12-11

**Authors:** Songyang Liu, Chenxi Cao, Yujun Zhang, Guangyu Liu, Weixia Ren, Yanqi Ye, Tiezheng Sun

**Affiliations:** 1 Arthritis Clinic and Research Center, People’s Hospital, Peking University, Beijing, 100044 People’s Republic of China; 2 Institute of Sports Medicine, Peking University Third Hospital, Peking University, Beijing, 100044 People’s Republic of China; 3 The Institute of Clinical molecular Biology and the Central Lab, Peking University, People’s Hospital, Peking University , Beijing, 100044 People’s Republic of China

**Keywords:** PI3K/Akt inhibitor, TNF-α, Cadherin-11, Osteoarthritis, Fibroblast-like synoviocytes

## Abstract

**Background:**

The Cadherin-11 and PI3K/Akt pathway are increasingly recognized as the potential therapeutic target of osteoarthritis (OA) synovitis. The study aimed to investigate the role of PI3K/Akt signaling pathway in the expression of Cadherin-11 and migration and invasive capacity of fibroblast-like synoviocytes (FLS) of OA patients under stimulation of TNF-α and to explore the effect of the PI3K/Akt inhibitor and Cadherin-11 antibody in the therapy of the collagenase-induced osteoarthritis (CIOA) mice.

**Methods:**

FLS were primarily cultured from synovium of osteoarthritic patients during total knee arthroplasty. Under the simulation of TNF-α, with or without PI3K/Akt inhibitor LY294002, Cadherin-11 expression was detected by real-time PCR and Western blot, as well as the migration and invasive capacity changes of OA FLS. Cadherin-11 antibody was injected intraarticularly or LY294002 was injected intraperitoneally in CIOA mice to evaluate the changes of synovitis score, cartilage damage, and Cadherin-11 expression.

**Results:**

TNF-α stimulation increased Cadherin-11 expression at mRNA and protein level in OA FLS and also increased the phosphorylation-dependent activation of Akt. PI3K inhibitor LY294002 attenuated TNF-α-induced overexpression of Cadherin-11 and decreased the invasive capacity of OA FLS. Intraperitoneal injection of PI3K inhibitor LY294002 could decrease the Cadherin-11 protein expression in synovium of CIOA mice, although it has no significant inhibitory effect on synovitis and cartilage damage. Intraarticular injection of Cadherin-11 antibody attenuated the synovitis and cartilage damage in the CIOA joints and decreased Cadherin-11 expression in the synovial lining.

**Conclusions:**

PI3K/Akt pathway was associated with TNF-α-induced activation of OA FLS, which may involve in the pathogenesis of osteoarthritis. Anti-Cadherin-11 therapy in CIOA mice could attenuate the pathological changes of OA joints.

## Background

Osteoarthritis (OA) is the most common form of arthritis and forms a major burden to health care [1, 2]. Previously, we have long considered it as a non-inflammatory degenerative joint disease, characterized by articular cartilage degradation. However, in recent years, many research papers indicate that osteoarthritis contains pathological changes of all structures within the joint, including the synovial inflammation, hyaline articular cartilage loss, and bony remodeling changes [3]. Synovial inflammation is increasingly recognized as contributing to the pathology during progression of osteoarthritis [4]. An arthroscopic study also suggests that synovitis occurs in 50% of patients with OA [5], and the synovial activation was associated with cartilage pathology [6]. Previous researches suggest that many inflammation cytokines, such as TNF-α and IL-1β, might participate in OA synovial inflammation [7, 8]. It has been assumed that inflammatory cytokines might act as triggers of the inflammatory cascade amplification in local synovitis in OA.

It has been proposed that fibroblast-like synoviocytes (FLS) are actively involved in chronic inflammatory reactions. The molecular mechanisms of their sustained activation might be contributed to inflammatory cascades. Cadherin-11 is a classical molecule that mediates hemophilic cell-to-cell adhesion in FLS, plays an important role in the development of the normal synovium lining layer of the joint [9–11], and has been identified to participate in the cartilage invasion except for its adhesive function [10]. Cadherin-11-deficient mice have a hypoplastic synovial lining, display disorganized synovial reaction to inflammation, and are resistant to inflammatory arthritis [12]. Our previous study demonstrated that Cadherin-11 expression in FLS was positively related to the degree of synovitis [13]. As a predominant inflammatory factor, TNF-α plays a vital role in the upregulation of Cadherin-11 in FLS. However, the underlying mechanism for the upregulation of Cadherin-11 expression in FLS by the stimulation of TNF-α was rarely investigated.

The study objective was to explore whether PI3K/Akt signaling pathway was involved in the TNF-α-induced upregulation of Cadherin-11, as well as the migration and invasive capacity of OA FLS. In the meantime, we aimed to examine the potential therapeutic effect with PI3K/Akt inhibitor and anti-Cadherin-11 antibody in collagenase-induced osteoarthritis (CIOA) mice.

## Materials and methods

### Isolation and culture of fibroblast-like synoviocytes

Synovial tissues were collected at the time of total knee arthroplasty (TKA) from 26 patients with primary OA in the Arthritis Clinic and Research Center, People’s Hospital, Peking University, Beijing, China. The diagnosis of OA fulfilled the criteria of the American College of Rheumatology (ACR) in 1985 [12]. The mean age of the osteoarthritic patients was 65.8 years old (range 55–76), 5 males and 21 females.

FLS were isolated by enzymatic dispersion of synovial tissue from the OA patients [14]. Briefly, synoviocyte suspensions were prepared from the synovial membranes after mincing and incubation with 1 mg/ml type I collagenase (Invitrogen, CA, USA) in low-glucose Dulbecco’s modified Eagle’s medium (DMEM) for 2–3 h at 37 °C. The cell suspensions were filtered through a 70-μm cell strainer (BD Biosciences, CA, USA), extensively washed, and placed in tissue culture dishes in DMEM supplemented with 10% (volume/volume, v/v) fetal bovine serum (FBS, Invitrogen, Australia), 100 U/ml penicillin, and 100 μg/ml streptomycin in a 37 °C, 5% CO_2_ incubator.

After the third passage, non-FLS cells completely disappeared from these culture systems, and the remaining cells were mainly FLS as previously described [15], which were proved by flow cytometry. FLS from the third to the sixth passages were chosen for the subsequent experiments.

Isolated FLS were seeded in six-well plates containing 3 ml DMEM supplemented with 10% FBS and grown to subconfluence of 80%. After starvation in the serum-free culture medium for 24 h, the regular medium was replaced and FLS were treated with TNF-α and/or PI3K/Akt inhibitor. For TNF-α treatment, each culture plate was treated by the recombinant human TNF-α (R&D Systems, Minneapolis, MN, USA) for 24 h at concentrations of 10 ng/ml. Control cultures containing no cytokines were grown in parallel. To evaluate the effect of PI3K/Akt on the FLS activation induced by TNF-α, FLS were pre-incubated with 10 μM LY2940002 (PI3K/Akt inhibitor) for 1 h before TNF-α stimulation. The inhibitor was diluted in dimethyl sulfoxide (DMSO), and control cells were pre-incubated with equivalent amounts of DMSO alone.

### Quantitative real-time PCR

Total ribonucleic acid (RNA) was obtained from OA FLS using TRIzol reagent (Invitrogen, Camarillo, USA) according to the instructions. After determining the spectral value with NanoVue Plus spectrophotometer (GE Healthcare, Piscataway, USA), 0.1 μg RNA was reversely transcribed into cDNA using SuperScript III First-Strand Synthesis System (Invitrogen).

Subsequently, qPCR was performed using MiniOpticon qPCR (Bio-Rad, Hercules, USA). The reaction mixture contained 2 μl cDNA, 6.4 μl pure water, 0.8 μl forward and reverse primer, and 10 μl SYBR qPCR Mix (QPS-201, Toyobo, Shanghai, China). The primer sequences for GAPDH were 5′-GTCTCCTCTGACTTCAACAGCG-3′ (forward) and 5′-ACCACCCTGTTGCTGTAGCCAA-3′ (reverse), and the primer sequences for Cadherin-11 were 5′-GATCGTCACACTGACCTCGACA-3′ (forward) and 5′-CTTTGGCTTCCTGATGCCGATTG-3′ (reverse).

The whole process consisted an initial activation step at 94 °C for 2 min followed by 40 amplification cycles: 94°Cfor 15 s (denaturation), 60 °C for 20 s (renaturation), and 72 °C for 30 s (extension). The specificity of the primers was verified by the melting curve analysis. Data were analyzed using the comparative threshold method (ΔΔCt), and the results were expressed as 2^−ΔΔCt^.

### Western blot

After treatment with TNF-α and PI3K/Akt inhibitor, total cell lysate was obtained by incubating the FLS monolayers with RIPA Kit (Solarbio, Peking, China). The protein concentration was determined using BCA protein assay kit (Solarbio, Peking, China). An identical amount of proteins (20 μg) was separated using 8% SDS-PAGE and blotted onto PVDF membranes (Bio-Rad, Hercules, USA). Blots were blocked for 1 h with 5% BSA in Tris-buffered saline (TBS) at room temperature and then incubated overnight at 4 °C with anti-Cadherin-11 (1:1500 dilution, Abcam, Cambridge, MA, USA), anti-Akt (1:2000 dilution), and anti-P-Akt (1:1000 dilution, Cell Signaling Technology, Beverly, MA, USA) antibodies. Subsequently, bound antibody was detected with horseradish peroxidase-labeled goat anti-rabbit IgG (1:1000 dilution, sc-2005, Santa Cruz, Dallas, USA) and visualized with an enhanced Chemiluminescence Kit (Thermo Scientific, Rockford, USA). Protein loading was checked and standardized by β-actin (anti-β-actin antibody, 1:2000 dilution; Proteintech, Chicago, USA). Quantification of the band intensity was performed using Image J software (National Institutes of Health, USA).

### Cell migration and invasion assay

2 × 10^4^ OA FLS in serum-free DMEM medium were seeded in the upper chamber of the 8.0-μm pore filter migration inserts (Transwell; Corning, NY, USA) in 24-well cell culture plates, with or without TNF-α. To evaluate the effect of PI3K/Akt on the migration and invasion capacity treated by TNF-α, FLS were pre-incubated with 10 μM LY294002 for 1 h before TNF-α stimulation for 24 h. The lower chamber contained DMEM medium with 10% fetal bovine serum (FBS). After incubation for 24 h, cells that had not penetrated the filter were removed from the top of the filter using cotton swabs, and the remaining cells were fixed in 4% (weight/volume, w/v) paraformaldehyde for 15 min, then stained with 0.1% (w/v) crystal violet, and counted. Values for migration were expressed as the average number of migrated cells bound per microscopic field (× 100). Three microscopic fields per membrane in triplicate experiments were counted.

For invasion assay, the migration inserts were coated with 20 μl 10% (v/v) Matrigel (BD Bioscience, San Diego, CA, USA) at 37 °C for 45 min. Then, 2 × 10^4^ OA FLS in serum-free DMEM medium were seeded likewise. After incubation for 24 h, cells that had not penetrated the filter were removed from the top of the filter using cotton swabs, and the remaining cells were fixed in 4% (w/v) paraformaldehyde for 15 min, then stained with 0.1% (w/v) crystal violet, and counted. Values for invasion were expressed as the average number of migrated cells on the underside of the filters per microscopic field (× 100). Three microscopic fields per membrane in triplicate experiments were counted.

### Induction of collagenase induced OA mice model

Thirty male C57/BL6 mice aged 8–12 weeks were randomly divided into five groups with six for each group: (1) control group, (2) OA + isotype IgG group, (3) OA + Cadherin-11 antibody group, (4) OA+ LY2940002 (PI3K/Akt inhibitor) group, and (5) OA + DMSO group.

Experimental OA mice model was induced as previously described [16]. In brief, the procedure involves intraarticular administration of 10 μl collagenase VII (containing 1 unit collagenase) (Sigma, St. Louis, MO) in the right knee on day 0 and day 2.

After 2 weeks of collagenase induction, 2 μg isotype IgG (ZSGB-BIO, China, ZDR-5001) or Cadherin-11 antibody (sc-30,314, Santa-Cruz, USA) was separately intraarticularly administered twice a week for 2 weeks. Then, the mice were euthanized with CO_2_, and the right knees were isolated and processed for histological examination.

After 2 weeks of collagenase induction, 1 mg LY294002 dissolved in 200 μl 8%DMSO diluted by PBS was inoculated intraperitoneally (i.p.) daily for 2 weeks. Two hundred microliters of 8%DMSO-PBS was inoculated i.p. daily for the control group. After 2 weeks, the mice were euthanized with CO_2_, and the right knees were isolated and processed for histological examination.

### Histological assessment of articular cartilage damage and synovitis score

Intact mouse knees were dissected away from the skin, fixed in 4% paraformaldehyde for 2 days and decalcified in 10% formic acid for approximately 1 week. Then, the specimens were embedded in paraffin and sections (4 μm) throughout the knee (five sections at 100 μm distance) were required for the HE, Safranin O/Green staining, and Cadherin-11 staining.

Krenn’s synovitis score on HE stained synovial membrane sections included the synovial hyperplasia graded by mean synovial lining layer thickness (1–3 scale, where 1 = mean of 1–2 cell layers, 2 = mean of 3–5 cell layers, and 3 = mean of > 5 cell layers), the density of stromal cells, and inflammatory infiltration of the sublining layer. This semiquantitative scoring method has been extensively validated.

Cartilage damage score was performed according to the OARSI scoring system on Safranin O/Green staining of knee joint sections, which was a modification from Chambers et al. [17]. The scoring system is recommended to apply to all four quadrants of the joint: medial tibial plateau (MTP), medial femoral condyle (MFC), lateral femoral condyle (LFC), and lateral tibial plateau (LTP). A score of 0 represents normal cartilage, 0.5 = loss of PG with an intact surface, 1 = superficial fibrillation without loss of cartilage, 2 = vertical clefts and loss of surface lamina (any % or joint surface area), 3 = vertical clefts/erosion to the calcified layer lesion for 1–25% of the quadrant width, 4 = lesion reaches the calcified cartilage for 25–50% of the quadrant width, 5 = lesion reaches the calcified cartilage for 50–75% of the quadrant width, and 6 = lesion reaches the calcified cartilage for > 75% of the quadrant width. Five sections were obtained and evaluated for each mouse. The cartilage damage index was expressed as summed score which can be combined for the entire joint or split out for MTP, MFC, LTP, or LFC separately.

For immunohistochemical staining of Cadherin-11, paraffin sections (4 μm) were routinely prepared, dewaxed with the endogenous peroxidase inactivated by 3% (v/v) H_2_O_2_, then incubated in citrate buffer (0.1 M, pH 6.0) at 98 °C for about 20 min. After blocking with 10% (v/v) BSA, sections were incubated with 1:100 anti-Cadherin-11 rabbit polyclonal antibody (Clone WNTID1, Invitrogen, Camarillo, USA) overnight at 4 °C. Parallel sections were incubated with a nonspecific isotope, and concentration matched antibody as negative control. After incubation with the primary antibody, sections were sequentially incubated with horseradish peroxidase-conjugate goat anti-rabbit immunoglobulin G (IgG) (Gene. Shanghai. China) for 60 min at room temperature and visualized with 3,3-diaminobenzidine (Solarbio, Peking, China). Finally, the sections were counterstained with hematoxylin.

As described previously, Cadherin-11 staining was scored semiquantitatively based on intensity [categorized as 0 (absent), 1 (weak), 2 (moderate), or 3 (strong)] and the percentage of positively stained area [scored as 0 (0–5% positive), 1 (6–25%), 2 (26–50%), 3 (51–75%), or 4 (> 75%)] in the lining layer and sublining layer [18]. The evaluator of the joints was blinded to the treatment groups.

### Statistical analysis

The statistical analysis was performed using SPSS Version 20.0 (IBM Corp, NY, USA). The normally distributed data such as the values from Western blot and qRT-PCR were expressed as mean ± SD and were analyzed using one-way analysis of variance (ANOVA). The abnormally distributed data like histological parameters of synovitis score and cartilage damage score of different groups were expressed as mean ± SEM and compared by the Kruskal–Wallis non-parametric test. The Mann–Whitney *U* test was used for direct comparisons between the two groups. *p* values less than 0.05 were considered significant (*p < 0.05, **p < 0.01, ***p < 0.001).

## Results

### Cadherin-11 upregulation by TNF-α was associated with PI3K/Akt pathway

**Fig. 1:**
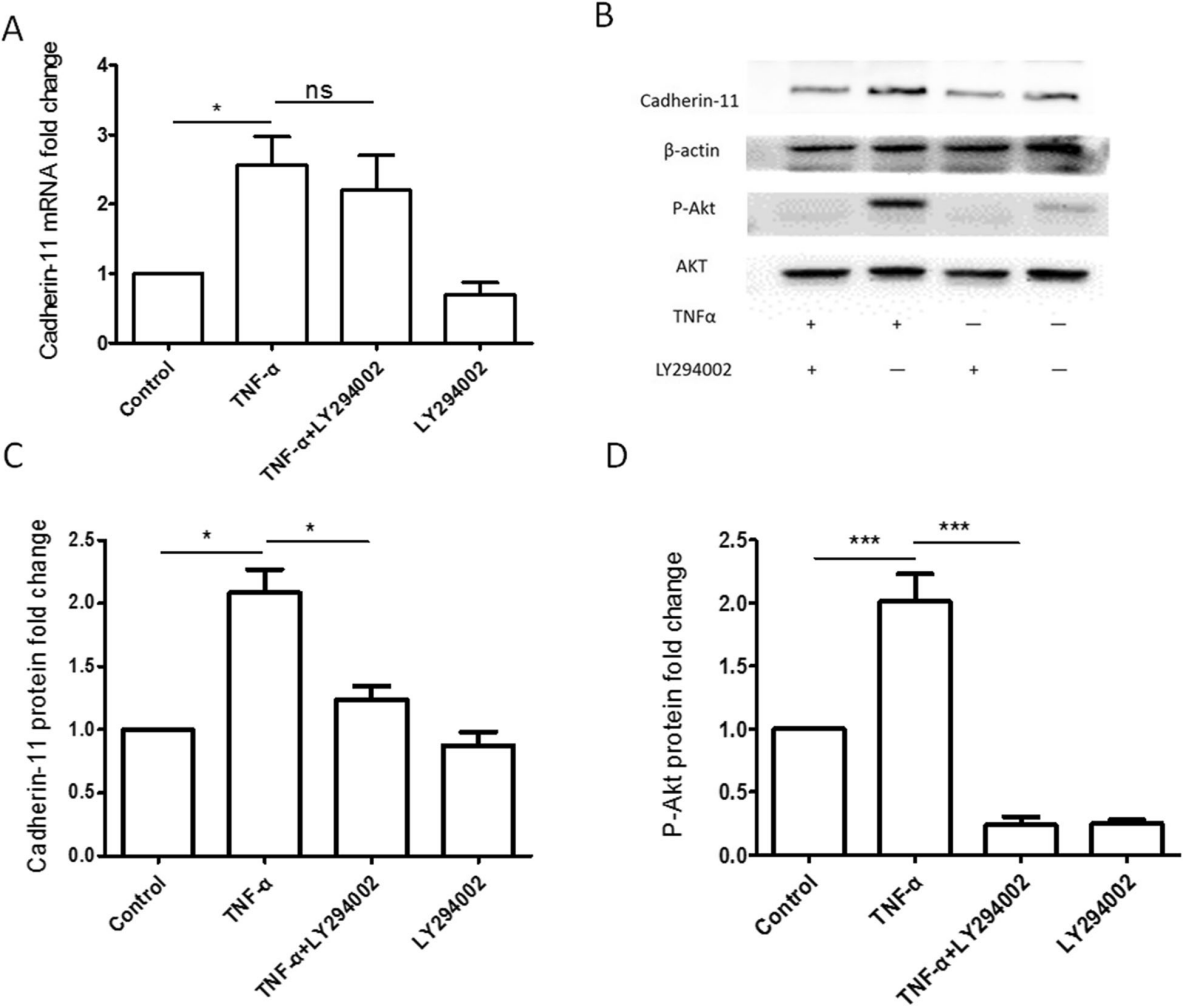
Expression of Cadherin-11 and P-Akt in OA FLS induced by TNF-α with or without PI3K inhibitor LY294002. **a** Real-time PCR showed that cadherin-11 mRNA increases with the stimulation of TNF-α in OA FLS (*p* < 0.05), but PI3K inhibitor LY294002 had no significant inhibitory effect (*p* > 0.05). **b** Western blot showed that expression of Cadherin-11 and phosphorylation of Akt (P-Akt) increased with the stimulation of TNF-α in OA FLS (*p* < 0.001), while PI3K inhibitor LY294002 had significant inhibitory effect (*p* < 0.001). **c** Quantification analysis of expression of Cadherin-11 protein. The amount of each protein was determined densitometrically and normalized to β-actin. **d** Quantification analysis of P-Akt. The amount of each protein was determined densitometrically and normalized to total Akt. The experiment was performed three times. Values were means ± SD, *p < 0.05, **p < 0.01, ***p < 0.001

Cadherin-11 was increased at mRNA and protein level in OA FLS with TNF-α stimulation compared to the control group (Fig. 1a, b). Also, TNF-α stimulation could increase the phosphorylation-dependent activation of PI3K/Akt (Fig. 1b, d, *p* < 0.05).

To explore the role of PI3K/Akt signaling pathway in expression of cadherin-11 induced by TNF-α in OA FLS, the specific PI3K inhibitor, LY294002, was used to pre-treat OA FLS before TNF-α stimulation. As shown in Fig. 1d, phosphorylation of Akt induced by TNF-α was significantly inhibited by LY294002 (Fig. 1d, *p* < 0.001). Expression of Cadherin-11 protein induced by TNF-α was significantly attenuated after incubation with LY294002 (Fig. 1b, c, *p* < 0.05). Increased expression of Cadherin-11 mRNA induced by TNF-α was slightly reduced by LY294002; however, it was not statistically significant (Fig. 1a, *p* > 0.05).

### PI3K inhibitor, LY294002, inhibits the migration and invasive capacity of OA FLS induced by TNF-α

**Fig. 2:**
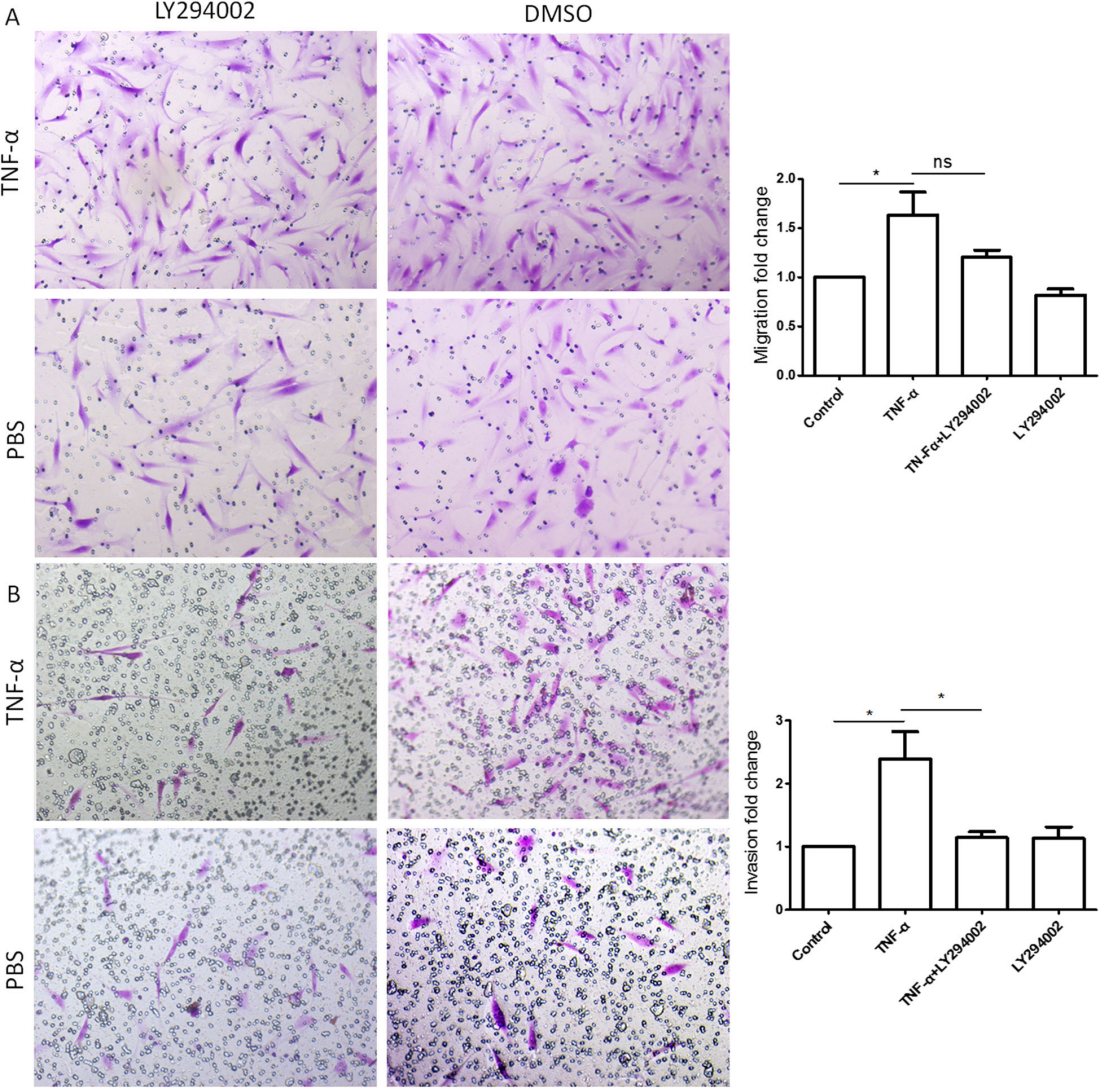
Effect of LY294002 on the migration and invasive capacity of OA FLS with stimulation of TNF-α. TNF-α increased migration (**a**) and invasive (**b**) capacity of OA FLS (*p* < 0.05). PI3K inhibitor, LY294002, could inhibit the invasive capacity of OA FLS induced by TNF-α (*p* < 0.05), but has little inhibitory effect on migration of OA FLS (*p* > 0.05). Five fields per filter were selected randomly and photographed. All experiments were repeated three times. Data are expressed as mean ± SD. *p < 0.05, **p < 0.01, ***p < 0.001

As shown in Fig. 2, TNF-α stimulation enhanced the migration and invasive capacity of OA FLS. PI3K inhibitor, 10 μM LY294002, inhibited in vitro invasion capacity of OA FLS stimulated by TNF-α (Fig. 2b, *p* < 0.05), but only slightly inhibited the migration capacity of OA FLS (Fig. 2a, *p* > 0.05). These results suggested that PI3K/Akt pathway might be involved in modification of the cellular invasiveness behavior of OA FLS.

### PI3K inhibitor and anti-Cadherin-11 therapeutics on synovial hyperplasia and cartilage damage in CIOA mice

**Fig. 3:**
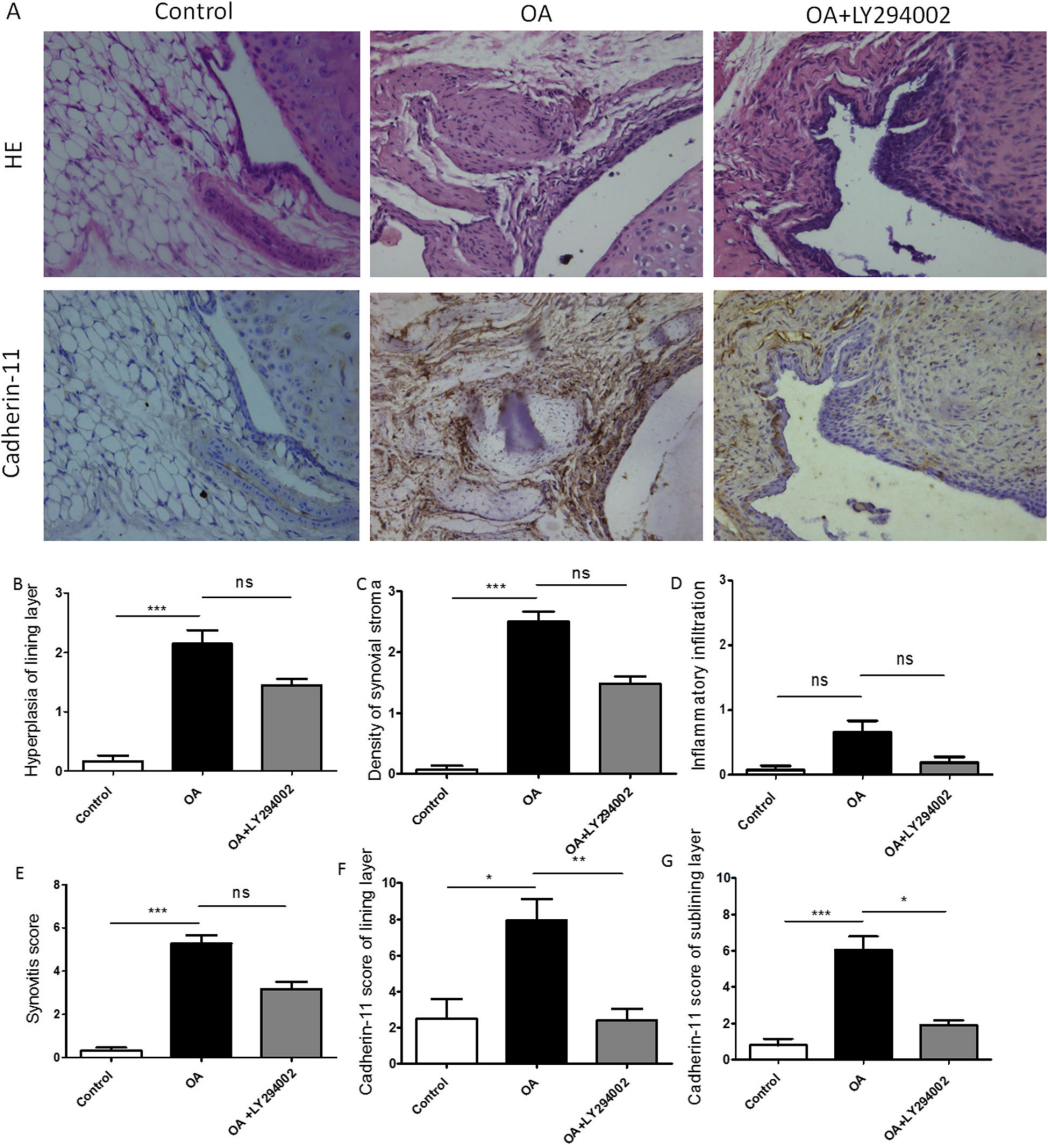
Effect of LY294002 on synovitis score and expression of Cadherin-11 in CIOA mice. Representative photomicrographs of HE staining and Cadherin-11 immunostaining of synovium in CIOA mice with or without PI3K inhibitor, LY294002. **a** PI3K inhibitor, LY294002, significantly decreased expression of Caherin-11 in synovial lining layer (**f**, *p* < 0.01) and sublining layer (**g**, *p* < 0.05) of CIOA mice; however, it has no obvious effect on synovial hyperplasia, inflammatory infiltration, and density of synovial stromal cells (**b**, **c**, **d**, *p* < 0.05) as well as the total synovitis score (**e**, *p* > 0.05) (*n* = 6 per group respectively; data presented as mean ± SEM; Kruskal–Wallis test followed by Mann–Whitney *U* test, *p < 0.05, **p < 0.01, ***p < 0.001)

**Fig. 4:**
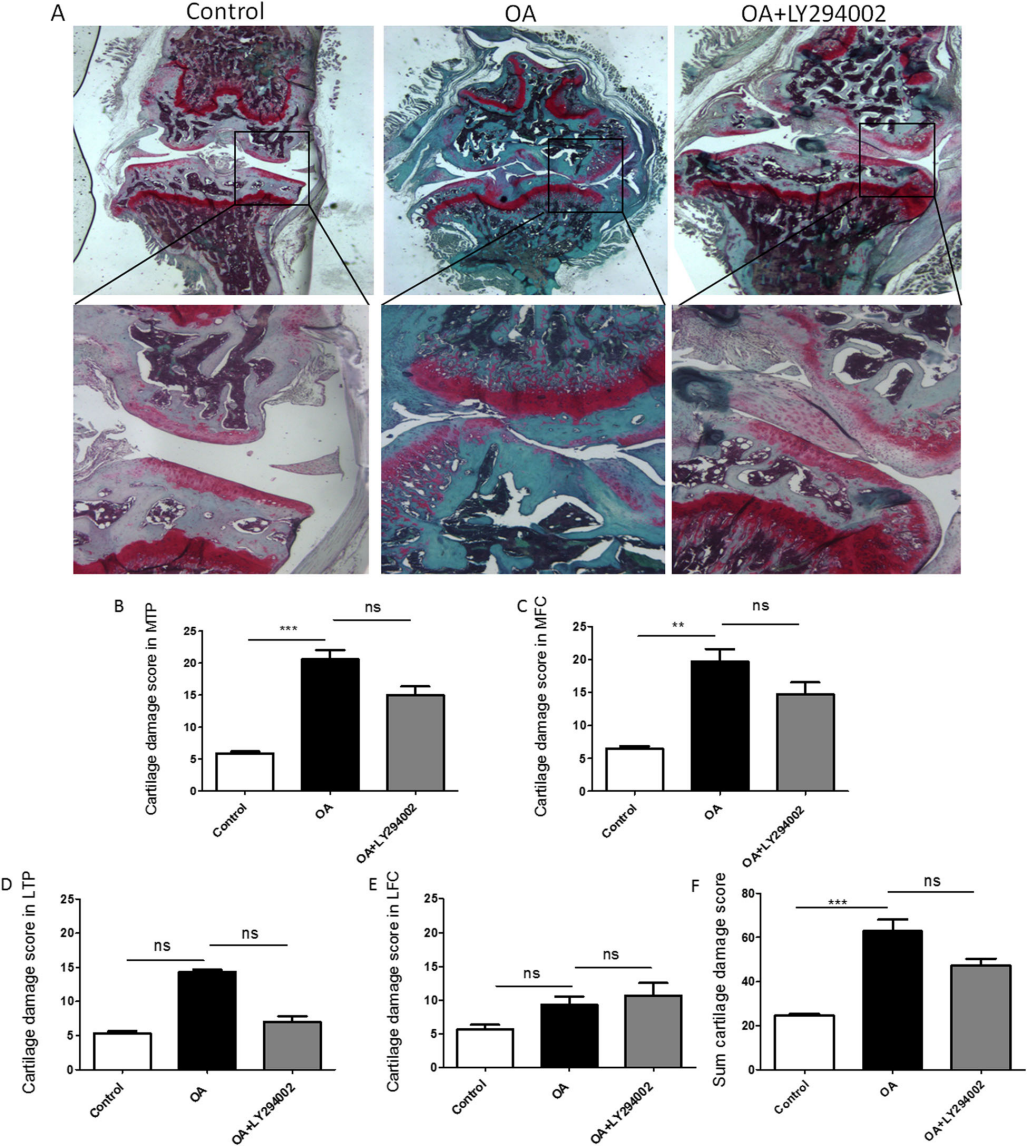
Effect of LY294002 on OARSI cartilage score in CIOA mice. Representative photomicrographs of Safranin O/Green staining of knee joints in l CIOA mice (**a**). LY294002 showed no significantly inhibitory effect on cartilage damage score in MTP (**b**, *p* > 0.05), MFC (**c**, *p* > 0.05), LTP (**d**, *p* > 0.05), LFC (**e**, *p* > 0.05), and summed cartilage damage score (**f**, *p* > 0.05) (*n* = 6 per group respectively; data are presented as mean ± SEM; Kruskal–Wallis test followed by Mann–Whitney *U* test, *p < 0.05, **p < 0.01, ***p < 0.001)

In the collagenase-induced OA mice, a clear thickening of the synovial lining layer was found compared to the control mice (Fig. 3a). As shown in Fig. 4a, digital image analysis showed that the cartilage damage mostly occurred in the medial tibial plateau (MTP) (Fig. 4b, *p* < 0.001) and medial femoral condyle (MFC) (Fig. 4c, *p* < 0.01).

After the treatment of LY294002, expression of Cadherin-11 in the synovial lining layer (Fig 3f, *p* < 0.01) and sublining layer in CIOA was significantly reduced (Fig. 3g, *p* < 0.05), but there was no significant change on synovial hyperplasia, sublining inflammatory infiltration, and density of stromal cells (Fig. 3b–d, *p* > 0.05). Either the total synovitis score did not show any significant difference (Fig. 3e, *p* > 0.05). The treatment of LY294002 showed no significant effect cartilage destruction in the four separate quadrants (Fig. 4b–d, *p* > 0.05) and the summed cartilage score (Fig. 4e, *p* > 0.05) in the CIOA model.

**Fig. 5:**
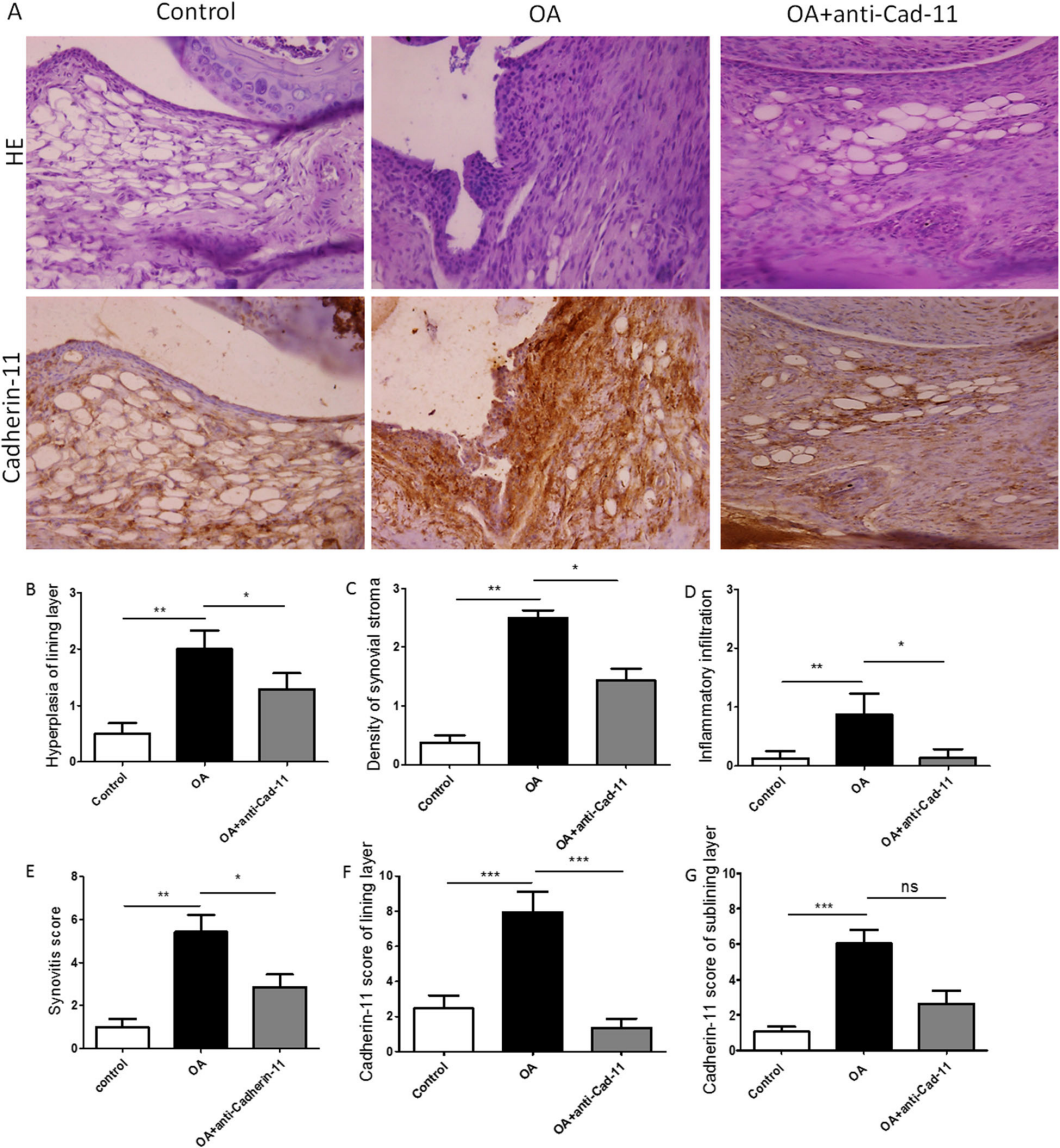
Effect of cadherin-11 antibody on synovitis score and expression of Cadherin-11 in CIOA mice. **a** Representative photomicrographs of HE staining and Cadherin-11 immunostaining of synovium in CIOA mice with or without cadherin-11 antibody. Cadherin-11 antibody has significant inhibitory effect on Krenn synovitis score (**e**, *p* < 0.05), including synovial hyperplasia (**b**, *p* < 0.05), inflammatory infiltration (**c**, *p* < 0.05), and density of synovial stromal cells (**d**, *p* < 0.05). Cadherin-11 antibody significantly decreased expression of Caherin-11 in the synovial lining layer (**f**, *p* < 0.001), but not in the sublining layer (**g**, *p* > 0.05) in CIOA mice. Anti-Cad-11 represents anti-Cadherin-11 antibody therapy group (*n* = 6, 9, and 9 per group respectively; data presented as mean ± SEM; Kruskal–Wallis test followed by Mann–Whitney *U* test, *p < 0.05, **p < 0.01, ***p < 0.001)

**Fig. 6:**
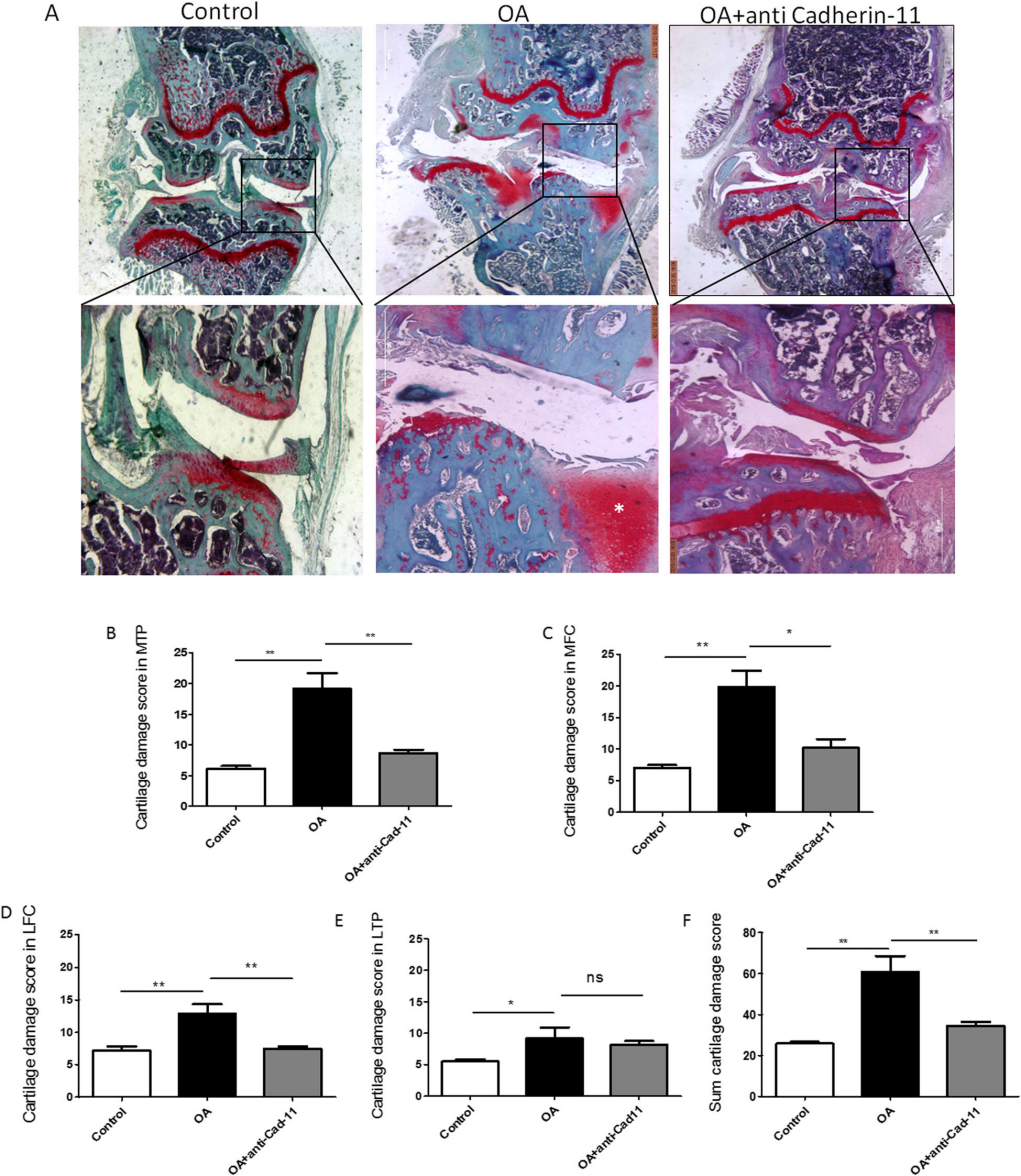
Effect of Cadherin-11 antibody on cartilage damage score in CIOA mice. Representative photomicrographs of Safranin O/Green staining of knee joints in CIOA mice (**a**). Cadherin-11 antibody showed significant inhibitory effect on cartilage damage score in MTP (**b**, *p* < 0.05), MFC (**c**, *p* < 0.05), LTP (**d**, *p* < 0.05), LFC (**e**, *p* < 0.05), and summed cartilage damage score (**f**, *p* < 0.05) (*n* = 6, 9, and 9 per group respectively; data presented as mean ± SEM; Kruskal–Wallis test followed by Mann–Whitney *U* test, *p < 0.05, **p < 0.01, ***p < 0.001)

In the Cadherin-11 antibody group, typical features of OA including synovial hyperplasia, sublining inflammatory infiltration, and density of stromal cells were significantly attenuated compared to the CIOA group (Fig. 5b–d, *p* < 0.05). The general synovitis score also decreased significantly after Cadherin-11 antibody treatment (Fig. 5e, *p* < 0.05). Expression of Cadherin-11 in the synovial lining layer in CIOA was reduced after treatment of Cadherin-11 antibody (Fig. 5f, g, *p* < 0.001). The cartilage damage score in MTP (Fig. 6b, *p* < 0.01), MFC (Fig. 6c, *p* < 0.05), and the summed score in the CIOA model was alleviated by injection of Cadherin-11 antibody (Fig. 6f, *p* < 0.01).

## Discussion

It has been proved that the PI3K/Akt signaling pathway can be activated following TNF-α exposure [19]. Feldman et al. has also demonstrated that PI3K/Akt pathway can be activated by several cytokines such as TNF-α in RA synoviocytes [20, 21]. In this study, the overexpression of Cadherin-11 in OA FLS after being treated with TNF-α was accompanied by the activation of P-Akt, which represents the persistent activation of PI3K/Akt pathway. However, when pre-treated with LY294002, the protein expression of P-Akt and Cadherin-11 in OA FLS was significantly reduced, although the inhibitory effects were not significant on Cadherin-11 mRNA level. In fact, from DNA to mRNA to protein expression, there are three levels of regulation including transcription, translation, and post-translation. Thus, the mRNA expression is only related to the transcription level, which cannot fully represent the whole process of regulation. Chang et al. [14] pointed out that the activation of Akt can cause the phosphorylation of GSK3β at the Ser9 site and thereby inhibiting its kinase activity. However, Vandooren et al. [15] reported that glycogen synthase kinase 3 beta (GSK-3β) stabilizes Cadherin-11 mRNA expression by β-catenin-independent pathway in prostate cancer and breast cancer cells. It also regulates the stability of the untranslated region of Cadherin-11 at post-transcriptional level via β-catenin-dependent pathway, which leads to a decrease in the expression level of Cadherin-11 protein. In combination with our results, the reason why Cadherin-11 did not show a significant decrease in mRNA level after treatment with LY294002 in OA FLS may be due to LY294002 causing the inhibition of Akt activation, which abates the inhibition of GSK-3β thus leading to the increased stability of Cadherin-11 mRNA, while the decrease of Cadherin-11 protein expression is caused by the regulation of transcriptional regulation of GSK3β through β-catenin-dependent pathway. Actually, the PI3K/Akt regulates a cascade of changes through its broad target proteins such as mTOR, NF-kB, GSK-3β, and p53 [22], which suggests that PI3K might be involved in post-transcriptional regulation of cadherin-11. So, we concluded TNF-α induced the increases of Cadherin-11 expression via PI3K/Akt pathway in OA FLS, which was consistent with the fact that PI3K/AKT pathway plays an important role in synovial inflammation [23].

PI3K/Akt might serve as a cross-link between OA FLS invasion and cartilage damage. The migration of FLS to the cartilage and bone has been considered a critical step in the erosion of joints in OA and RA. It is well established that pro-inflammatory cytokines, such as IL-1β and TNF-α, constitute key mediators of FLS migration and invasion [24]. We found that inhibition of PI3K/Akt could significantly decrease the invasive capacity of OA FLS under the stimulation of TNF-α, but the inhibition of migration capacity was not significant. This implies that FLS following the inflammatory reaction might result in the invasion of FLS and destruction of cartilage. It is known that OA FLS was required to penetrate matrix in the invasion assay compared with the migration assay. Therefore, the reduced invasive ability may be related to the decreased secretion of MMP3 or MMP9 after inhibition of PI3K/Akt signaling. In addition to its role in FLS, PI3K/Akt activation can induce a series of downstream signaling pathways and target protein, including NF-kB, GSK-3β, and p53. [25, 26]. confirmed that the activation of P-Akt and NF-kB signaling pathways plays an important role in the inflammatory factor-mediated secretion of MMPs. And these may explain why only the invasion capacity has changed while migration capacity has not.

Given the important roles uncovered for PI3K in promoting inflammation, many studies have investigated the effects of pharmacological inhibition of PI3K isoforms in animal models of inflammatory joint disease. The PI3Kγ inhibitors AS605240 and TASP0415914 reduced the symptoms of collagen-induced arthritis (CIA) [27, 28]. ZSTK474, a pan-class I PI3K inhibitor, was also found to improve inflammation and disease progression in RA animal models [29]. However, few studies focused on the application of PI3K/AKT inhibitors on the OA treatments. Cartilage erosion in the collagenase-induced osteoarthritis (CIOA) model was ultimately driven by the remodeled synovial lining and direct effect of synoviocytes. In the CIOA mice, we demonstrated that the collagenase injection resulted in osteoarthritis-like changes including higher degree of synovitis, more pronounced chondral defects occurring in the medial tibio-femoral compartment, and not completely mineralized osteophytes. Our results showed that there was no significant effect on synovitis score and cartilage damage score in CIOA mice injected i.p. with PI3K inhibitor, LY294002. Li et al. injected LY294002 with 20 mg/kg intraperitoneally for the treatment of rheumatoid arthritis in rats. After 3 weeks, the symptoms were relieved and synovial hyperplasia and inflammatory cell infiltration improved [30]. Although PI3K inhibitors have great efficacy, studies have shown that they may be at the expense of increased toxicity. Hu et al. also reported weight loss and dry skin in mice with ovarian cancer after intraperitoneal injection of LY294002 at 100 mg/kg [31]. Extensive inhibition of PI3K is likely to cause significant toxicity because PI3K I is critical for normal physiological processes such as glucose homeostasis and immune responses. Therefore, based on the consideration that LY294002 toxicity may cause death in mice, we prudently selected 50 mg/kg as the final therapeutic dose. However, the difference in pathogenesis and characteristics between the carcinoma and OA imply the distinct dose and duration of therapy of PI3K inhibitors, which needs further research.

We already know that for most of the in vivo processes involved, the effects of class I PI3K inhibition are usually partial. Specifically, the inhibition of PI3Kγ can be blunt recruitment and activation of innate immune cells, which is not complete; the inhibition of PI3Kδ prevents a normal antibody response, but some other antibodies are made; inhibition of PI3Kδ and β can inhibit antibody-dependent activation of neutrophils and macrophages, but bacterial uptake and killing are relatively unscathed. On the other hand, macrophages are important constituent cells of the synovial lining layer. In addition to regulation of synovial inflammation, macrophages can also mediate the process of cartilage damage. After clearance of macrophages in CIOA synovial inflammation, cartilage destruction was significantly alleviated. Synovitis and cartilage destruction in the CIOA model of S100A9^−/−^ mice were significantly improved [32] Although we confirmed that LY294002 can inhibit the ability of OA FLS invasion, its role on macrophages is not clear. It may explain that LY294002 did not improve synovitis and cartilage destruction in the CIOA model. In addition, since multiple signaling pathways that regulate OA FLS are independent of each other and balance with each other, a single block of a pathway may not be a good inhibitor of hyperplasia and activation of OA FLS, resulting in less than ideal results. Thus, the robust and redundant processes that underlie the inflammatory response may allow an opportunity to inhibit class I PI3K-dependent processes to a level in which a significant decrease of Cadherin-11 expression is possible but alleviation of the pathology is not complete. Furthermore, the inflammatory cytokines in the OA pathological process trigger a variety of downstream signaling pathway.

The protective effect of anti-Cadherin-11 therapy has been demonstrated convincingly in a range of arthritis models. Lee et.al [29] found that anti-Cadherin-11 displays moderate amelioration of established K/BxN serum transfer arthritis. Kou et al. [33] indicated that blocking Cadherin-11 partially reversed the TMJ inflammatory pain and estradiol-potentiated proliferation of synovial lining cells. This study was the first to investigate the anti-Cadherin-11 therapeutics in the CIOA mice, which mostly resembled the synovium inflammation and cartilage damage in the knee OA patients. In the study, not only the synovitis was partially attenuated after the Cadherin-11 antibody injection, but also the condition of cartilage damage was improved to some extent, especially in the MTP and MFC. Overexpression of Cadherin-11 in the synoviocytes might be positively related to the increased invasive capacity of FLS and articular cartilage destruction while anti-Cadherin-11 antibody significantly alleviates the pathology changes.

There were some limitations of our study. The role of other cytokines, such as IL-1 and IL-6, on activating OA FLS through the PI3K/Akt signaling pathway has not been investigated. Moreover, CIOA mice models are not the same as human OA, and the applicability of low-toxicity PI3K/Akt inhibitor or Cadherin-11-targeted therapy to other OA models and human OA still needs to be evaluated.

## Conclusions

PI3K/Akt pathway was associated with TNF-α-induced activation of fibroblast-like synoviocytes in OA, which may be involved in the pathogenesis of osteoarthritis. Anti-Cadherin-11 therapy in CIOA mice could attenuate the pathological changes of OA joints, although PI3K/AKT inhibitor’s influence was not complete. Further clarification of the mechanism underlying the upregulation of PI3K/Akt induced by TNF-α should be examined with promising results in the field of potential alternative treatments of OA. More detailed studies should provide additional insights into the mechanisms relevant to the development and progression of osteoarthritis.

## Data Availability

The datasets used and/or analyzed during the current study are available from the corresponding author on reasonable request.

## Abbreviations

ANOVA: Analysis of variance
cDNA: Complementary DNA
CIOA: Collagenase-induced osteoarthritis
DMEM: Dulbecco’s modified Eagle’s medium
FBS: Fetal bovine serum
FLS: Fibroblast-like synoviocytes
GAPDH: Glyceraldehyde-3-phosphate dehydrogenase
GSK-3β: Glycogen synthase kinase 3 beta
MMP: Matrix metalloproteinase
OA: Osteoarthritis
PBS: Phosphate-buffered saline
PI3K: Phosphatidylinositol 3-kinase
qRT-PCR: Quantitative real-time polymerase chain reaction
RA: Rheumatoid arthritis
SD: Standard deviation
SEM: Standard error of the mean
TNF-α: Tumor necrosis factor α
